# Lactating Adenoma in Pregnancy: Report of Three Cases

**DOI:** 10.34172/aim.28751

**Published:** 2025-02-01

**Authors:** Julija Cvetković, Maja Jovičić Milentijević, Nikola Živković, Miodrag Đorđević, Jelena Grujović, Goran Radenković

**Affiliations:** ^1^Center for Pathology, University Clinical Center Niš, 18000 Niš, Serbia; ^2^Clinic for Endocrine Surgery, University Clinical Center Niš, 18000 Niš, Serbia; ^3^Department of Histology and Embryology, Faculty of Medicine, University of Niš, 18000 Niš, Serbia

**Keywords:** Adenoma, Breast, Case reports, Lactation, Pregnancy

## Abstract

**Background:**

Lactating adenoma is an infrequent benign stromal breast tumor mostly seen during pregnancy and lactation. This report seeks to enhance the literature by presenting three cases diagnosed with this condition, while also highlighting its histological subtypes, immunohistochemical characteristics, and differential diagnoses.

**Case Presentation:**

In all three patients, a circumscribed, painless, and mobile mass was found in the breast in the third trimester of pregnancy. Pathohistological examination revealed hyperplastic lobules with glandular formations in back-to-back arrangements showing more or less abundant hobnailing phenomena with intraluminal eosinophilic secretions and inconspicuous myoepithelial cell layer separated by fibrovascular stroma. The immune profile showed positive reaction for cytokeratin 14 and p40 markers, indicating the presence of myoepithelial cells and distinguishing these cases from other breast lesions.

**Conclusion:**

Pathohistological and immunohistochemical analysis should be performed to differentiate these lesions from other benign lesions and malignant tumors.

## Introduction

 Lactating adenoma is a benign tumor of the breast that is mostly seen during pregnancy and lactation.^1^ Most commonly, they affect primigravidae between 20 and 35 years of age in the last trimester of pregnancy or lactation.^2,3^ Lactating adenomas are usually small in size (about 3 cm), painless, solid, well-circumscribed, and mobile nodule masses that tend to grow slowly.^4-7^ Pathophysiologically, it has not been fully investigated whether these adenomas arise *de novo* or in already existing hyperplastic lesions.

 Some theories may refer to pre-existing fibroadenoma, tubular, or lobular adenoma under hormonal changes.^2^ However, lack of mediator complex subunit (MED) 12 exon 2 mutations, commonly seen in fibroadenomas, weakens this hypothesis.^8^ Others suggest that they develop *de novo* due to heightened levels of estrogen. On the other hand, pathohistological examination and differentiation of lactating adenoma from fibroadenoma could be challenging.^9^ Lactating adenomas are distinct from fibroadenomas, as they primarily consist of epithelial elements with minimal stromal tissue and lack the myoepithelial cell layer.^10^ Clinically, lactating adenomas present similarly to fibroadenomas, with a note that they typically regress after cessation of breastfeeding and can occur in succeeding pregnancies.^11,12^ Generally, these masses represent a diagnostic challenge because elevated hormone levels during pregnancy promote neoangiogenesis and glandular tissue proliferation, while on the other hand, the stromal component is reduced.^10,13^ Ultrasound features suggestive of lactating adenomas include a spherical hypo/isoechoic lesion with posterior enhancement and sharp edges, which may sometimes contain infarcted cystic fields.^14^ Although lactating adenomas are generally considered benign, there have been rare instances of their coexistence with invasive carcinomas.^15-17^ The golden standard in diagnosing lactating adenomas is a biopsy followed by pathohistological analysis.^18^ These cases emphasize the need for thorough evaluation of breast nodules during pregnancy, as the incidence of breast cancer in pregnant patients is increasing and timely diagnosis is crucial for effective patient management, which should not be postponed until after childbirth.

## Case Report

 Here, we present three cases of females with lactating adenomas. All three patients had a similar clinical presentation, which included a change in their breasts in the third trimester of pregnancy. A circumscribed, painless, and mobile mass was discovered in all three women. There was no change in the skin, such as depression, discoloration, or dimpling. No axillary lymphadenopathy was detected. They were not on any medications and had no allergies. A history of smoking was denied, and they drank alcohol occasionally. Ultrasonographically, these neoplasms appeared in two patients as spherical masses with parallel orientation, well-defined edges, iso-to-hypoechoic echo pattern, and posterior enhancement. However, an irregular, echogenic, vascularized change in the lactiform canal was observed on ultrasound of the third patient. All three patients had a score of 3/4 in Breast Imaging-Reporting and Data System (BIRADS). Surgical removal of the changes was indicated, followed by pathohistological analysis. The pregnancies of all three patients ended favorably, with the birth of three healthy children. The patients showed no recurrence or complications during the one-month postoperative follow-up.

 A gross examination of the specimen from patient 1 revealed a fragment of fatty and glandular tissue, white in color, measuring 55 × 53 × 15 mm. On the cross-section, there was adipose tissue interspersed with bands of glandular tissue, with hemorrhage in the surrounding area. Microscopic examination revealed a well-defined proliferation of hyperplastic lobules that were densely packed, with layers of epithelial and myoepithelial cells divided by thin, delicate stromal tissue. The cuboidal and some hobnail-shaped cells lined the glandular formations with abundant intraluminal eosinophilic secretions. These cells lacked cytological atypia, their nuclei were small and round, and they had granular to vacuolated cytoplasm. Other micromorphological findings included typical lobular hyperplasia on the resection margins of the specimen.

**Figure 1:**
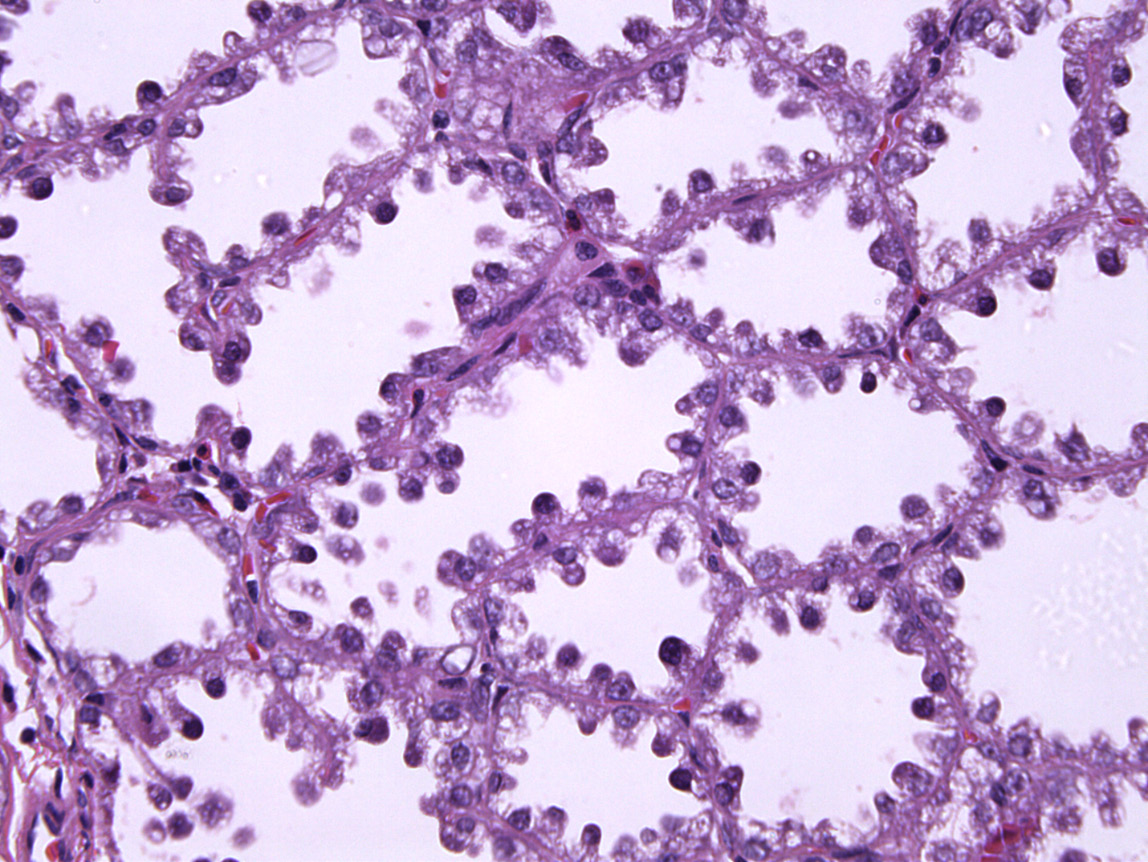
Prominent Nuclear Hobnailing with Bulbous Nuclear Projections into the Lumen and an Inconspicuous Myoepithelial Cell Layer (Patient 2) (H&E × 400)

 The surgical specimen from patient 2 grossly consisted of two tissue fragments representing the fatty and glandular white to greyish breast tissue, measuring 30 mm and 35 mm. Microscopically, the preparation showed greatly expanded lobules of variably sized glands in a back-to-back arrangement. The hyperplastic lobules were separated by delicate fibrovascular septae. The closely packed glands comprised prominent nuclear hobnailing with bulbous nuclear projections into the lumen and an inconspicuous myoepithelial cell layer (Figure 1). However, the eosinophilic secretion in the lumen of the glands was scant. The hobnail-shaped cells were not atypical and had no pathological mitotic figures. Their cytoplasm was granular and vacuolated, and the nuclei were small and round with variably prominent small, pinpoint nucleoli. Other pathological findings of the specimen included chronic periductal and intralobular mastitis with focal exacerbation.

**Figure 2:**
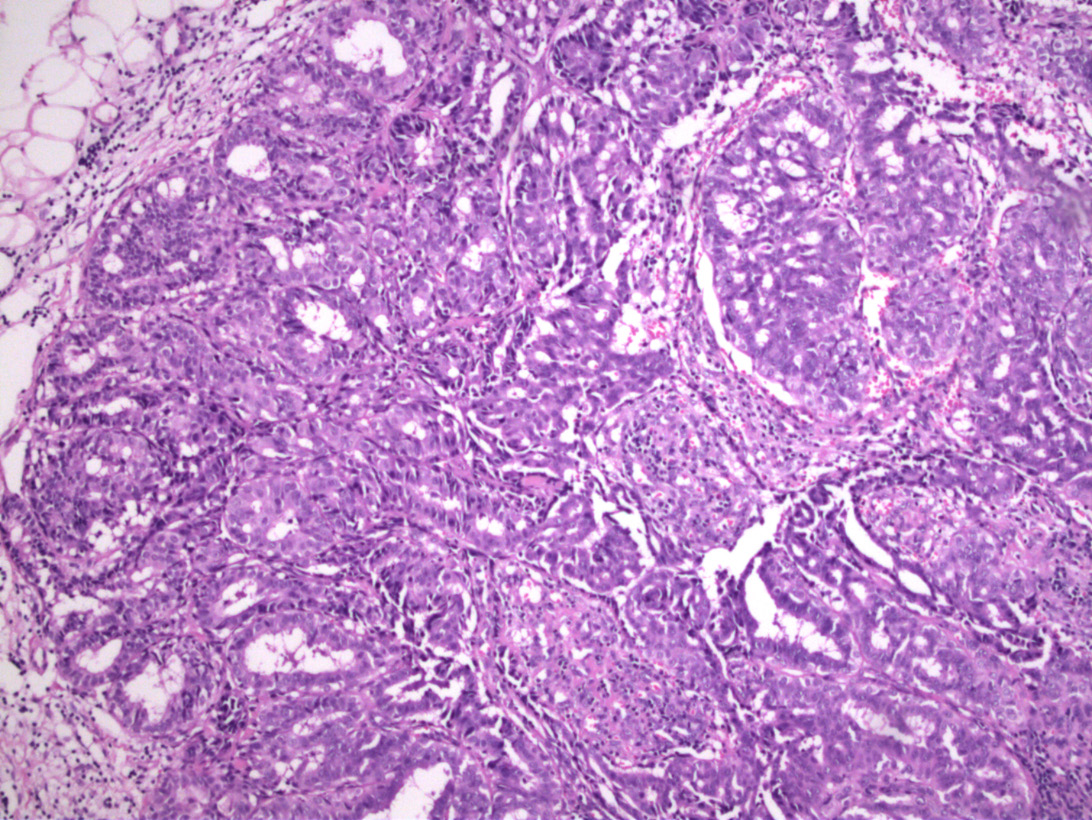
Lobules Separated by Slightly Thicker Strands of Intervening Stroma. Glands consisted of cuboidal and myoepithelial cells in mostly tubular histological patterns, with a smaller percentage of cribriform and solid patterns. glandular cells were oval to round, showing mild cytological atypia with rare mitotic activity. Cytoplasm was basophilic to granular with small, round, or elongated nuclei and prominent nucleoli. Hobnail-shaped cells and secretion into the lumen were scarce (patient 3) (H&E × 100)

 Gross examination of the specimen from patient 3 revealed two tissue samples of greyish-white color measuring 109 mm and 5 mm. Micromorphological examination of the preparation revealed well-circumscribed lobular hyperplasia with slightly thicker strands of intervening stroma. The glands consisted of cuboidal and myoepithelial cells in mostly tubular histological patterns, with a smaller percentage of cribriform and solid patterns. Glandular cells were oval to round, showing mild cytological atypia with rare mitotic activity. Their cytoplasm was basophilic to granular with small, round or elongated nuclei and prominent nucleoli. Hobnail-shaped cells and secretion into the lumen were scarce (Figure 2). In the surrounding adipose and fibrous tissue, nonspecific chronic inflammatory infiltrate was found.

**Figure 3:**
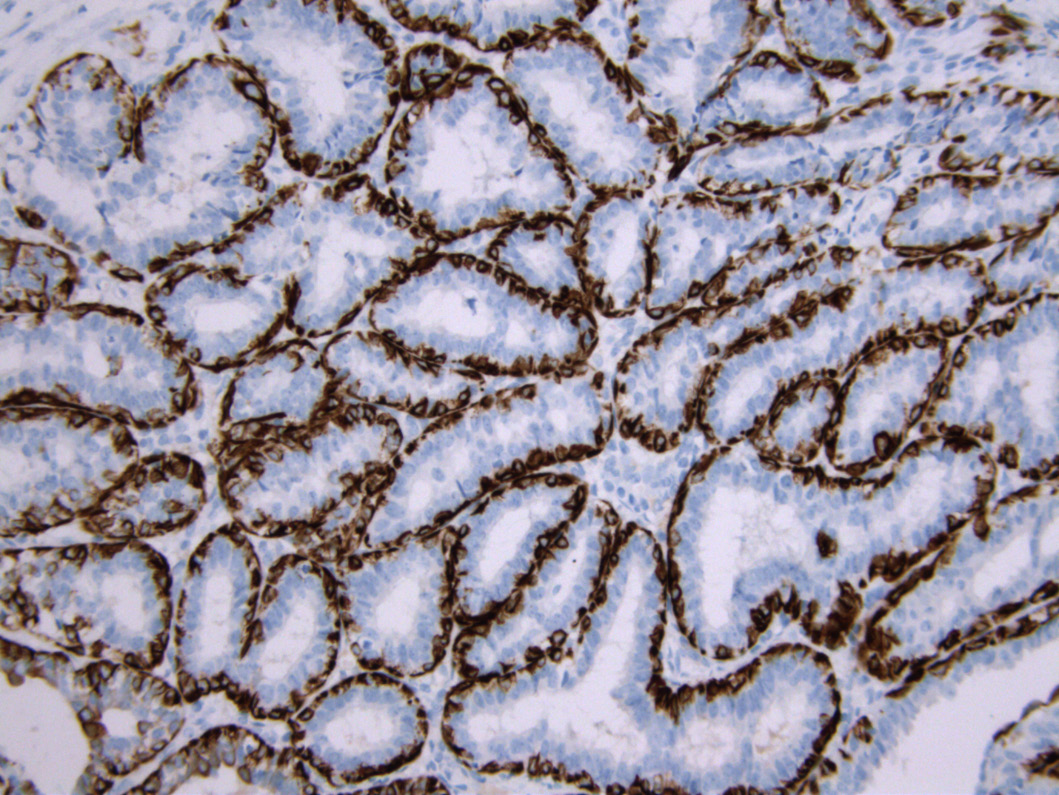
CK14 Immunoreactivity Present in the Myoepithelial Cell Layer of the Glandular Formations (CK14 immunohistochemistry × 200)

**Figure 4:**
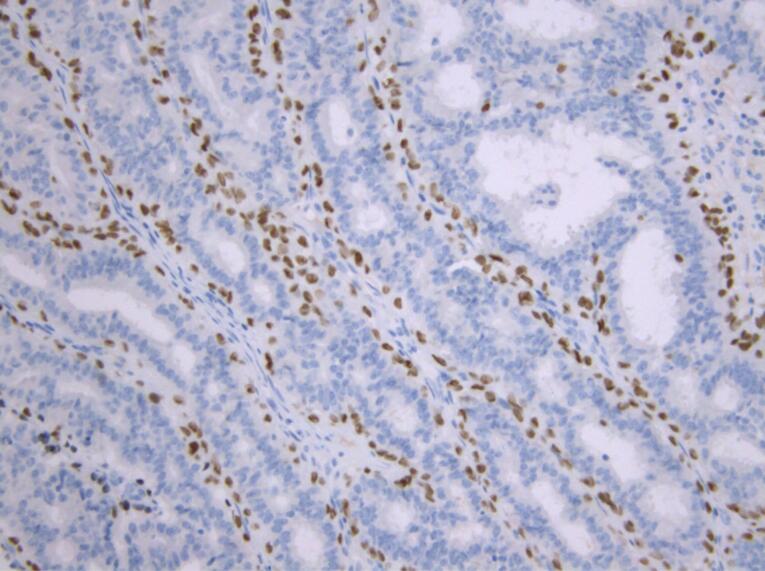
p40 Immunostain Positive in the Myoepithelial Cell Layer of the Glandular Formations (p40 immunochemistry × 200).

 In order to establish a definitive diagnosis and differentiate lactating adenoma from other breast diseases, an immunohistochemical analysis was performed. The immune profile showed a positive reaction for cytokeratin (CK) 14 and p40 markers, which indicated the presence of myoepithelial cells and distinguished these cases from other breast lesions (Figures 3 and 4).

## Discussion

 During pregnancy, hormones can cause various changes in the breasts, including the appearance of palpable masses, which further complicates both physical and radiological examination.^19^ Most lumps in pregnancy and lactation include lactating adenomas, fibroadenomas, and galactoceles.^20,21^ Given the fact that 3% of biopsied breast tissue samples during pregnancy and lactation are malignant tumors, any rapidly growing solid breast mass should be biopsied.^22,23^ On ultrasound, malignant changes can sometimes be masked by benign changes, especially if the mass has irregular borders, as in one of our patients. Changes of this type are usually well-defined solid, oval and lobular masses, around 3 cm in diameter.^24^ A homogeneous, hypoechoic appearance followed by posterior enhancement constitute the main characteristics of these lesions.^25^ Although most lactating adenomas tend to involute on their own, diagnosing them can be complex and surgical therapy may be necessary to rule out malignancy, particularly if they grow in size.^26^

 Pathohistologically, they consist of a well-defined proliferation of densely packed hyperplastic lobules, featuring layers of epithelial and myoepithelial cells segregated by scanty stromal tissue. Cells lining the glands are cuboidal or hobnail-shaped, they possess small round nuclei and clear vacuolated cytoplasm. Normally, the cells do not show signs of cytological atypia and can bear a resemblance to pregnancy-like changes.^27,28^ Despite this, histological analysis of one patient’s specimen showed signs of mild atypia, rare mitotic figures, and a smaller number of hobnail-shaped cells with intraluminal secretion. Lactational changes, in addition to those seen in lactating adenomas, can also appear focally in other breast lesions that are not associated with pregnancy, such as fibroadenomas.^29,30^ When the histological presentation is characteristic, discerning a lactating adenoma from a fibroadenoma usually does not present any difficulties. Fibroadenomas that develop extensive lactational change may exhibit converging characteristics with lactating adenomas. Fibroadenomas represent benign biphasic neoplasms composed of both glandular and stromal components of terminal duct lobular unit, while lactating adenomas primarily consist of epithelial component with conspicuous lactational changes. Generally, the presence of the stromal component can be enough to make a difference between a lactating adenoma and a fibroadenoma with lactational changes.^31^

 Furthermore, some malignant neoplasms can pose a greater challenge in histological differentiation. The main distinctive mark of secretory carcinoma is the presence of vacuolated, foamy cytoplasm of tumor cells and abundant intracellular and extracellular mostly eosinophilic secretions. Distinction between these two entities may be hindered when the secretory carcinoma is well differentiated, with only mild cytological atypia and indicative secretion.^32^ It is essential to review the patient’s history and medical background in such ambiguous situations. Secretory carcinoma primarily affects younger individuals and is not linked to pregnancy.^33^

## Conclusion

 Lactating adenoma requires a comprehensive evaluation to rule out malignancy, including physical examination, imaging, and histological findings. A thorough, individual approach is essential for every patient experiencing changes in the breast during pregnancy or lactation. This is crucial because delayed detection of malignancies in these specific conditions can lead to a worse prognosis and lower survival rates for women. Surgical therapy should be always performed when there is a family history of breast cancer, previous breast malignancy, or unclear and atypical radiological findings. Regarding the pathohistological evaluation of such cases, one should be careful and thorough to avoid overdiagnosing and declaring a benign condition malignant. Because of the specificity of these cases and doubtful micromorphological findings, immunohistochemistry needs to be performed to distinguish and confirm the benign nature of the condition.
